# Supplementary material for: Hybrid Immunity Improves the Immune Response after the Fourth COVID-19 Vaccine Dose in Individuals with Medical Conditions Predisposing to Severe COVID-19
Source: Vaccines (Basel). 2024 Feb 27;12(3):247. doi: 10.3390/vaccines12030247 (PMC10975123; doi:10.3390/vaccines12030247)

Supplementary material

Supplementary-Table S1. Number of risk group classifications per individual in the study cohort of participants with medical conditions predisposing to severe Covid -19 (n=488)

Number of risk group classifications	Number of individuals
**0**	38
**1**	56
**2**	135
**3**	110
**4**	75
**5**	31
**6**	34
**7**	7
**8**	2

Supplementary-Figure S1. Spike protein specific IgG antibody concentrations (full length spike protein; SFL-IgG and receptor binding domain; RBD-IgG) with geometric mean with 95% confidence interval shown one month after the fourth Covid-19 vaccine dose in participants with chronic kidney disease (CKD) and/or organ transplant. One participant can be in more than one group. IgG antibody concentrations are given as binding antibody units (BAU)/ml.

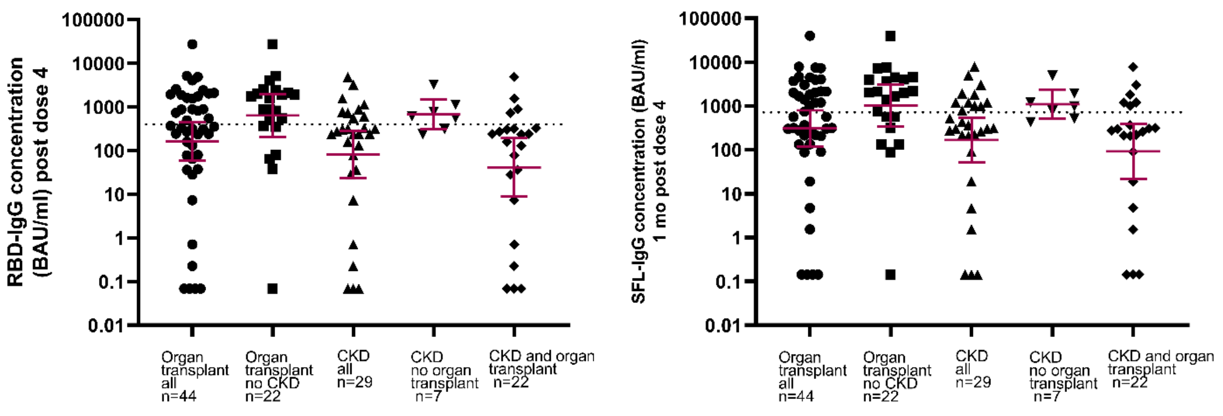

Supplementary-Figure S2. The correlation between RBD-IgG and SFL-IgG antibody concentrations (BAU/ml) and neutralizing antibody titers against wild type (WT) and Omicron BA.1 SARS-CoV-2 strains in samples taken one month after third (control, n=27) or fourth Covid-19 vaccine dose in participants with chronic kidney disease (with and without organ transplant) (n=20), chronic kidney disease and organ transplant (n=16), immunosuppressive therapy (n=20) and no predisposing medical factors affecting the risk of Covid-19 (no risk group, n=17). Spearman correlation coefficients (ρ) are shown.

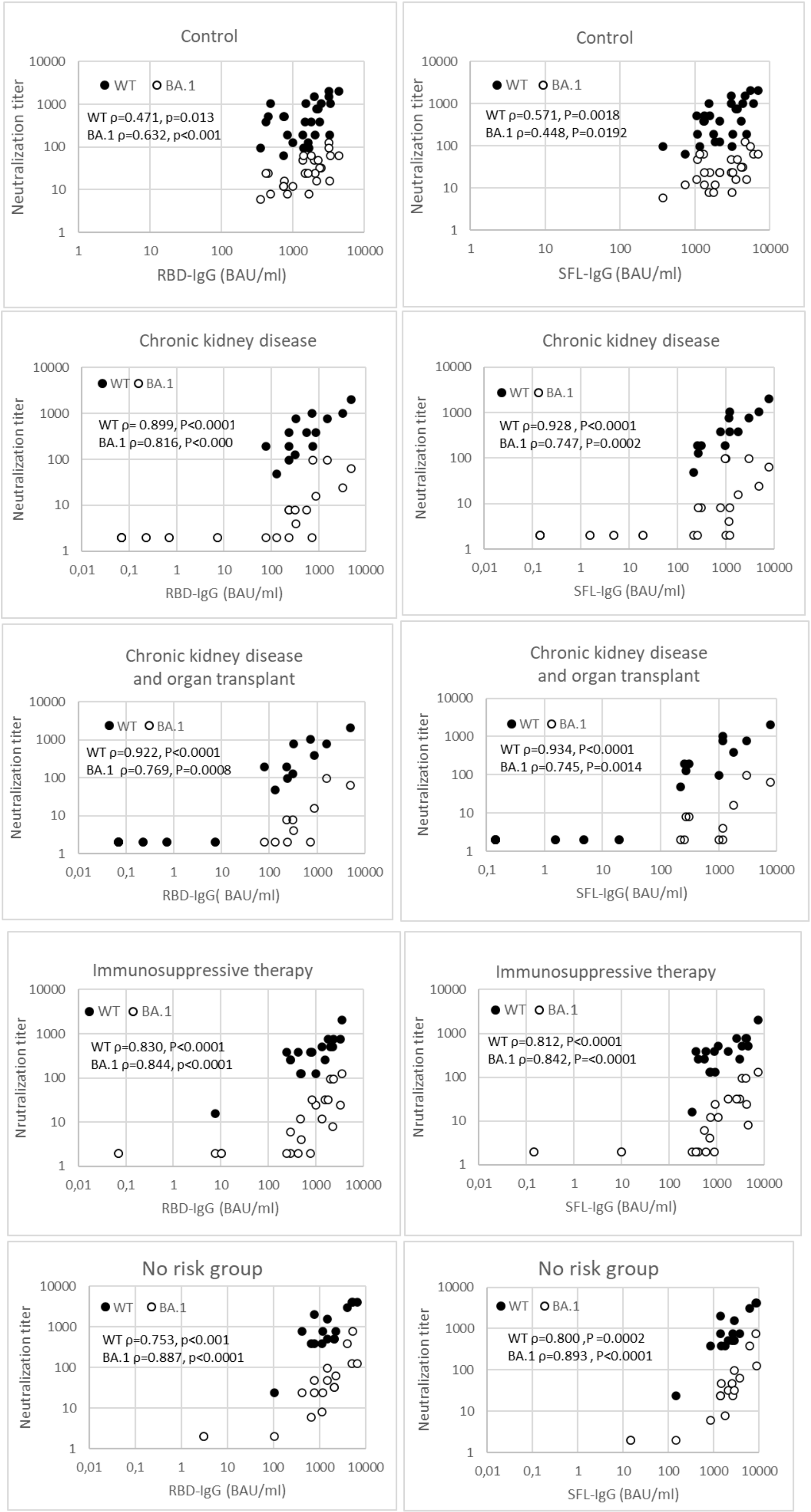

Supplement: Supplementary file 1 [file vaccines-12-00247-s001.zip › vaccines-2834514-supplementary.pdf]
